# The value of multimodal neuroimaging in the diagnosis and treatment of post-traumatic stress disorder: a narrative review

**DOI:** 10.1038/s41398-025-03416-1

**Published:** 2025-06-20

**Authors:** Hanyi Zhang, Yining Hu, Yiying Yu, Zheng Zhou, Yueru Sun, Chang Qi, Lihua Yang, Hua Xie, Junran Zhang, Hongru Zhu

**Affiliations:** 1 Mental Health Center, West China Hospital, Sichuan University, Chengdu, China; 2 Med-X Center for Informatics, Sichuan University, Chengdu, Sichuan China; 3 West China School of Public Health, Sichuan University, Chengdu, China; 4 Children’s National Hospital and Center for Neuroscience, Washington, DC USA; 5 College of Electrical Engineering, Sichuan University, Chengdu, Sichuan China

**Keywords:** Neuroscience, Psychiatric disorders

## Abstract

Post-traumatic stress disorder (PTSD) is a delayed-onset or prolonged persistent psychiatric disorder caused by individuals experiencing an unusually threatening or catastrophic stressful event or situation. Due to its long duration and recurrent nature, unimodal neuroimaging tools such as computed tomography (CT), magnetic resonance imaging (MRI), positron emission tomography (PET), and electroencephalography (EEG) have been widely used in the diagnosis and treatment of PTSD for early intervention. However, as compared with an unimodal approach, a multimodal imaging approach can better capture integrated neural mechanisms underlying the occurrence and development of PTSD, including predisposing factors, changes in neural activity, and physiological mechanisms of symptoms. Moreover, a multimodal neuroimaging approach can aid the diagnosis and treatment of PTSD, facilitate searching for biomarkers at different stages of PTSD, and explore biomarkers for symptomatic improvement. However, at present, the majority of PTSD studies remain unimodal, while the combination of multimodal brain imaging data with machine learning will become an important direction for future research.

## Introduction

Posttraumatic stress disorder (PTSD) is defined as the emergence or prolonged existence of psychiatric symptoms resulting from one’s encounter with an exceptionally threatening or catastrophic event. As described in the DSM-5, symptoms include recurrent re-experiencing of the trauma, avoidance of trauma-related stimuli, trauma-induced negative changes in mood and cognition, and increased vigilance [1]. In the United States, physical and sexual assaults, accidents, and fires are commonly reported traumatic events that can precipitate post-traumatic stress disorder. The lifetime prevalence rates for these events are 52 and 50%, respectively. Globally, accidents and injuries are the most frequently reported traumas, with a lifetime prevalence rate of 36%. The likelihood of developing PTSD is influenced by both the nature of the traumatic event and gender. For instance, the probability of developing PTSD following a physical assault is 2% for males and 22% for females [2]. According to the China Mental Health Survey (CMHS), the lifetime prevalence of PTSD is approximately 0.3%, with a six-month prevalence of around 0.2%. The prevalence is consistent at approximately 0.2% among men and women, showing no significant difference across sexes. Furthermore, the prevalence across different age groups ranges from 0.1–0.3%, with no notable sex differences [3].

PTSD is characterized by a protracted clinical course, a high propensity for relapse, and frequent psychiatric comorbidities, making its diagnosis and treatment challenging once symptoms manifest. Ongoing debates within the academic community regarding the underlying principles and mechanisms of PTSD also pose challenges for diagnosis and treatment. Therefore, the development of efficient and accurate diagnostic methods for PTSD, along with the enhancement of prognostic evaluation and the promotion of personalized treatment strategies, has emerged as a critical and urgent clinical challenge in the management of PTSD.

With advancements in neuroscience and imaging technologies, neuroimaging has emerged as an increasingly valuable tool for addressing diagnostic and therapeutic challenges in PTSD management. Current neuroimaging research indicates that various functional neural systems, including fear learning, threat monitoring, executive function, emotion regulation, and contextual processing, are pivotal in the pathophysiology of PTSD. These systems are involved in a wide range of structural and functional changes in the brain, including the amygdala, hippocampus, and prefrontal lobes [2]. Common brain imaging modalities include computed tomography (CT), magnetic resonance imaging (MRI), positron emission tomography (PET), and electroencephalography (EEG), these mental imaging methods have been widely used in the clinical diagnosis of PTSD and to assist in the formulation of treatment plans, but they all have limitations. To address these limitations, researchers and clinicians increasingly turn to multimodal data fusion techniques to present neuroimaging data in multiple dimensions, aiming to achieve a more comprehensive understanding of brain mechanisms.

Multimodal neuroimaging refers to an integrative technological approach that combines multiple imaging modalities, including structural magnetic resonance imaging (sMRI), functional magnetic resonance imaging (fMRI), magnetoencephalography (MEG) and so on, to comprehensively assess both structural and functional characteristics of the brain. It helps to understand the brain structure and function alterations in PTSD [4], differentiate between individuals with PTSD and those without psychiatric conditions [5], and evaluate the effectiveness of PTSD treatments. Multimodal neuroimaging is also used to identify biomarkers and mechanisms related to treatment outcomes [6–8]. It is increasingly used in diagnosing and treating mental health conditions.

Given this context, a systematic review and synthesis of the current achievements and demonstrated value of multimodal neuroimaging in PTSD diagnosis and treatment is both timely and essential. In this article, we first provide an overview of current perspectives on the neurophysiological mechanisms underlying PTSD. Subsequently, we summarize the existing challenges in PTSD diagnosis and treatment, and elucidate how multimodal neuroimaging approaches have addressed these issues, thereby highlighting the unique advantages and clinical value of this technology in PTSD diagnosis and treatment. In the final section, we critically examine the current limitations in multimodal neuroimaging research and propose potential directions and methodologies for future investigations and clinical applications.

## Neurobiological mechanisms of PTSD

### The HPA axis and fear learning and extinction

The hypothalamic-pituitary-adrenal (HPA) axis is a complex system involved in the stress response. In times of stress, the amygdala activates the sympathetic nervous system, leading to the release of adrenaline and noradrenaline, which then activate the HPA axis [9]. Extensive research has demonstrated that PTSD patients frequently exhibit dysregulation of cortisol levels and the HPA axis function, primarily characterized by reduced cortisol concentrations. However, it should be noted that some studies have failed to establish a significant correlation between HPA axis dysregulation and PTSD symptomatology. Despite variations in results, HPA axis dysregulation is widely recognized as a significant factor in PTSD [10, 11].

Another key mechanism in PTSD involves impaired fear learning and extinction. Fear learning includes conditioning and generalization: trauma-exposed individuals may link neutral stimuli to traumatic responses, triggering acute stress. PTSD patients further generalize fear by associating new neutral stimuli with stress [12]. Fear extinction, a safety learning process, requires recognizing safe environments. Studies show PTSD patients exhibit heightened fear responses, increased dACC activation during fear learning, and reduced vmPFC activation during extinction. Post-extinction, decreased amygdala and vmPFC activation to threats indicate impaired safety learning [13, 14]. Similar patterns occur in PTSD-induced healthy individuals [15]. Neuroimaging highlights the anterior hippocampus (aHPC) in fear processing, with PTSD patients showing fear generalization. A multimodal study (f-GBCr and d-GBCr) found aHPC-PFC connectivity negatively correlates with hypervigilance [16], suggesting PTSD patients have impaired fear regulation, leading to heightened vigilance.

The activation of the HPA axis is modulated by limbic system structures, particularly the amygdala, which plays a crucial role in fear conditioning and extinction processes. Furthermore, cortisol has been shown to facilitate fear extinction learning, thereby establishing a neurobiological link between HPA axis function and the mechanisms underlying fear acquisition and extinction [17–19]. Studies show that individuals with PTSD have heightened amygdala activation in response to fear stimuli, with increased fear responses linked to higher plasma ACTH levels [20]. This suggests that HPA axis dysregulation is associated with impaired fear inhibition. Exposure to severe trauma activates the amygdala, triggering the HPA axis and causing stress responses. Severe trauma can lead to fear conditioning without repeated pairing with neutral stimuli [21]. As a result, related neutral stimuli can repeatedly activate relevant brain regions, potentially leading to neural habituation in the prefrontal areas [22, 23]. This reduces the prefrontal cortex’s control over the limbic system and HPA axis, enhancing HPA feedback mechanisms and causing dysregulation. This dysregulation may result in exaggerated responses to certain neutral stimuli, increased fear, and difficulties in fear extinction and regulation. These neurophysiological mechanisms may contribute to the development and progression of PTSD.

### Dysregulation of multiple brain networks

Recent advancements in neuroimaging have shown that brain regions do not work in isolation but rather interactively, especially during complex cognitive tasks that require coordinated activity across networks. In the case of PTSD, there has been a shift towards studying brain network abnormalities, particularly focusing on the triple network model which includes the default mode network (DMN), salience network (SN), and central executive network (CEN). The DMN, which involves the posterior cingulate cortex, precuneus, medial prefrontal cortex, and hippocampus, plays a role in self-referential cognition, emotional regulation, and memory retrieval [24]. PTSD patients often exhibit reduced DMN connectivity [25, 26] and altered topological properties, such as decreased nodal degrees (except in the precuneus) [25], though pediatric PTSD patients exhibit increased degree centrality [27]. Multimodal neuroimaging studies have shown hyperconnectivity between the right medial prefrontal cortex and right inferior temporal cortex in PTSD patients with adverse childhood experiences (ACE) [28]. Additionally, functional changes in the precuneus and structural alterations in regions like the precuneus, inferior parietal lobule, and cingulate cortices have been identified through multimodal neuroimaging studies using DTI, fMRI, and sMRI [29]. PTSD patients show impaired connectivity in the DMN [30], leading to reduced integration and increased segregation. This can contribute to changes in self-concept, disrupted self-referential processing, and dissociative symptoms. The Salience Network, which includes the anterior insula, dorsal anterior cingulate cortex, and amygdala, is involved in monitoring internal and external stimuli and switching between the DMN and Central Executive Network (CEN). In PTSD, there is enhanced connectivity in the SN, potentially causing increased vigilance and somatic symptoms [31]. The CEN, which includes the dorsolateral prefrontal cortex and posterior parietal cortex, is important for executive functions. In PTSD, there is reduced connectivity in the CEN [26, 32], particularly in cases related to ACE with hypoconnectivity in the lateral prefrontal cortex and white matter [28], leading to cognitive impairments.

The triple network model focuses on both intra-network changes and inter-network dysregulation. In healthy individuals, DMN and CEN have an anti-correlated relationship, with the DMN supporting internal awareness and the CEN facilitating goal-directed tasks, while the SN mediates switching between these networks. In PTSD, this balance is disrupted [33]. Increased connectivity between the DMN and CEN may reflect a compensatory mechanism, reallocating resources to restore DMN activation at the expense of CEN function [34]. Meanwhile, increased SN-CEN connectivity [35] and decreased SN-DMN connectivity [36] suggest impaired top-down regulation by the CEN and disrupted switching between networks. Studies using DTI and fMRI during emotional Stroop tasks have linked SN and CEN abnormalities to white matter damage, potentially underlying symptoms like hypervigilance and attentional deficits [37]. Individuals with heightened fear arousal and avoidance show increased SN activation but insufficient CEN activation, leading to dysregulated SN-CEN coupling and compensatory hyperconnectivity.

The triple network model provides valuable insights, but it does not fully explain all PTSD symptoms. The visual network, including the visual cortex, occipital lobe, and lingual gyrus, is important for visual processing and is linked to hyperarousal, intrusive symptoms, and PTSD severity. Studies have shown increased activity in the visual cortex at rest in PTSD patients, as well as heightened activity in the left middle frontal gyrus and precuneus, which may contribute to visual symptoms like flashbacks [38, 39]. Functional abnormalities in the visual cortex [40], hyperactivation of the visual network have also been observed and the left middle occipital gyrus showed increases in nodal global efficiency and nodal degree [41], which are strongly associated with the visual symptoms manifested in PTSD patients. Research using structural and functional imaging techniques has demonstrated how changes in specific brain regions can impact PTSD symptoms. For example, a multimodal neuroimaging study by Harnett et al. found increased gray matter properties in the anterior temporal lobe, fusiform face area, and visual cortex were associated with acute stress disorder and PTSD symptom severity [42]. Other studies have shown that structural covariance networks in the ventral visual stream are linked to PTSD symptom severity, particularly intrusive and nightmare symptom [43]. These findings suggest that the visual network plays a significant role in hyperarousal and intrusive symptoms in PTSD.

Recent studies have also linked traumatic memory in PTSD to functional and structural abnormalities in the sensorimotor network (SMN) [44]. PTSD patients often fail to update memory representations with new information, a process dependent on the SMN and its coordination with other networks. Extreme and unpredictable stress can activate limbic regions such as the amygdala, triggering defensive responses. However, ineffective or absent defensive actions disrupt sensorimotor feedback, generating prediction errors that impair subcortical metabolism and function. This disrupts the transfer of information between sensorimotor and associative cortices, leading to hyperconnectivity within the SMN and preventing memory updating. As a result, traumatic memories are re-experienced as sensory and behavioral fragments. A multimodal meta-analysis found reduced SMN connectivity [4], impairing the perception of the “here and now,” while hyperconnectivity between the SMN and DMN was associated with memory re-experiencing [45], blurring temporal perception and contributing to traumatic re-experiencing. Overall, PTSD involves large-scale functional and structural dysregulation within and between multiple brain networks, collectively influencing symptom development and progression.

**Fig. 1:**
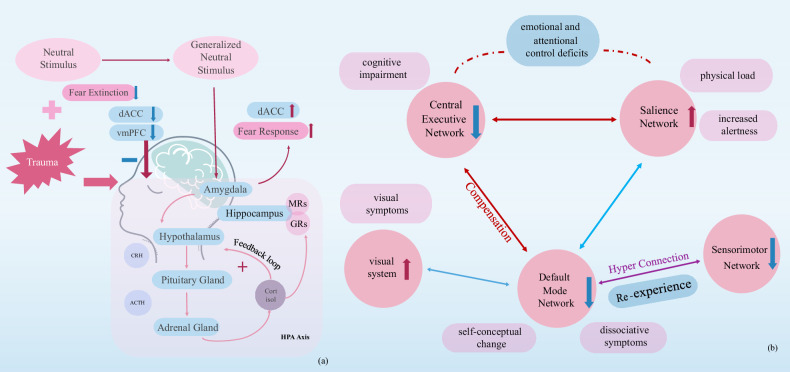
Neurobiological mechanisms of PTSD. **a** Due to the overwhelming intensity of traumatic experiences, neutral stimuli can elicit fear conditioning and activate the amygdala and HPA axis without requiring repeated pairing with aversive stimuli. This recurrent activation induces ventromedial prefrontal cortex habituation, impairing its regulatory control over the HPA axis and limbic system. The resulting cortisol feedback enhancement and HPA axis dysregulation underlie PTSD patients’ heightened fear responses and impaired extinction. The dorsal anterior cingulate cortex (dACC) modulates these processes, showing increased activation during fear responses and deactivation during extinction. **b** PTSD reflects widespread brain network dysregulation. Intra-network alterations include reduced connectivity in CEN, DMN, and SMN, alongside increased SN connectivity and visual network hyperactivation. Inter-network alterations are characterized by enhanced CEN-SN and CEN-DMN connectivity, decreased DMN-SN coupling, DMN-SMN hyperconnectivity, and reduced DMN-visual system connectivity, collectively reflecting extensive neural network impairment in PTSD.

The HPA axis-fear learning/extinction model and the multiple network theory provide complementary insights into the neurophysiological mechanisms of PTSD, drawing from psychiatric neuroimaging (Fig. 1). Multimodal neuroimaging has been crucial in advancing these perspectives by allowing for the simultaneous study of functional and structural connectivity to uncover dysregulation within and between networks. Compared to using just one type of imaging, multimodal approaches offer a more comprehensive understanding of how intricate brain networks contribute to PTSD symptoms and severity. Therefore, multimodal neuroimaging should continue to be a key component in future research on the neurobiological mechanisms of PTSD.

## Major brain imaging methods investigating PTSD

Before delving into the application of multimodal neuroimaging in PTSD diagnosis and treatment, it is crucial to first analyze the existing neuroimaging modalities, their roles in managing PTSD, and their strengths and weaknesses. This thorough examination will establish a strong basis for understanding the enhanced capabilities and clinical possibilities of multimodal neuroimaging techniques.

### MRI

Magnetic Resonance Imaging (MRI) is a technique that utilizes magnetic fields to generate images of bodily activity. It enables multimodal analysis, with Structural Magnetic Resonance Imaging (sMRI) offering detailed three-dimensional structural images for visualizing structural changes, functional Magnetic Resonance Imaging (fMRI) illustrating functional brain changes, and diffusion Magnetic Resonance Imaging (dMRI) mapping brain changes by measuring water molecule alterations. sMRI provides detailed structural images, fMRI visualizes brain function changes, and dMRI describes microstructural brain features by mapping axonal connections through water molecule measurements. Perfusion Weighted Imaging (PWI) primarily assesses tissue microvascular perfusion and blood flow to examine functional changes in brain tissue.

#### fMRI

Functional Magnetic Resonance Imaging (fMRI) is the primary method in cognitive neuroscience neuroimaging, which relies on MRI to assess changes in oxygenation dynamics induced by neuronal activity. By detecting alterations in the functional connections within the neural networks of brains, fMRI has been extensively applied to investigate PTSD.

Task state and resting state are two distinct functional states of the brain. The task state pertains to brain activity during cognitive tasks such as memory, emotion, and movement, while the resting state denotes brain activity when not engaged in cognitive tasks, and during a relaxed and awake state. Task fMRI captures brain activity during specific cognitive tasks, whereas resting state fMRI (rs-fMRI) records brain activity during relaxation without task engagement. Both task fMRI and rs-fMRI are extensively utilized in investigating brain mechanisms associated with PTSD.

In PTSD-related research, fMRI is crucial for investigating the neurophysiological mechanisms of symptoms such as emotional [18, 19, 22], fear and trauma memory [23–25, 27], and intrusive symptoms [28, 30] also utilized to discover biomarkers for diagnosis [26] and treatment [31, 32, 34]. In general, fMRI offers precise spatial resolution for studying brain neurofunction in PTSD research. Despite its widespread use, fMRI has limitations. Firstly, its temporal resolution is constrained by the slow blood oxygen response function. Secondly, fMRI captures numerous neuronal activity signals, with interpretations heavily reliant on task paradigm design. This reliance can hinder the accurate distinction between individualized functions and intrinsic neuromodulation, potentially leading to confusion between excitatory and inhibitory processes. Compared to EEG, which can identify subtle differences in different cognitive processes through microcomponents, fMRI signals have difficult in quantification. Because the hemodynamic response is very sensitive to the size of the activation region, leading to spatiotemporal changes in the BOLD signal due to the spatial density of neural representations. Cognitive processes entail a collaboration of different activities, with some having sparse neural representations while others significantly impact the hemodynamic response. The sensitivity of the hemodynamic signal to activated regions allows for the recognition of signal changes even in small areas, complicating the direct inference of specific roles of these regions in cognitive processes based solely on fMRI imaging results [46]. Integrating fMRI and EEG data offers a promising avenue for examining brain activity variations with high temporal and spatial resolution. Lastly, fMRI data can serve as a valuable tool for data-driven and definitive biological profiling. Nonetheless, the inconsistent results of such profiling [47] indicate the instability of using fMRI alone to elucidate the diverse mechanisms of PTSD in the brain. The instability may stem from individual differences, sample variability, and the limitations of fMRI in identifying consistent brain patterns among PTSD patients sharing the same biotype. Complementing fMRI with other modalities of brain imaging data, particularly multimodal approaches, may offer more reliable insights into the heterogeneous mechanisms underlying PTSD symptoms.

#### sMRI

Structural magnetic resonance imaging (sMRI) is commonly utilized to evaluate regional structural characteristics of the brain. Current sMRI studies on PTSD have predominantly examined alterations in GMV. However, there is a growing amount of research focusing on metrics such as cortical thickness and morphometrics [35]. Numerous sMRI investigations have revealed changes in GMV or density in PTSD patients, particularly in regions within the limbic-prefrontal circuit such as the anterior cingulate gyrus and insular cortex [48, 49], the ventral ACC and orbital frontal cortex [50, 51], as well as the frontal and occipital lobes [52], limbic and paralimbic cortex [53], and medial prefrontal cortex [49, 54]. Structural MRI findings elucidate alterations in brain region structures in individuals with PTSD, providing insights into the underlying mechanisms of PTSD onset and progression from a structural perspective, thereby enhancing our understanding of PTSD mechanisms.

In addition, sMRI holds promise in identifying biomarkers to differentiate individuals with PTSD from those without the disorder. Gong et al. utilized brain structural features extracted from sMRI data in a machine learning approach to distinguish between PTSD patients, trauma-exposed controls (TECs), and HCs [55]. Their findings revealed that alterations in gray and white matter enabled accurate differentiation between PTSD survivors and healthy controls (91% accuracy), as well as between TECs and healthy controls (76% accuracy). Moreover, only gray matter changes could distinguish PTSD patients from TECs (67% accuracy). These results underscore the potential of sMRI in discriminating among individuals with PTSD, TECs, and healthy individuals. It should also be noted that GMV alone has limitations in effectively distinguishing PTSD patients from HCs. Zhang et al. demonstrated that a multimodal dataset combining GMV, amplitude of low-frequency fluctuation (ALFF), and regional homogeneity (ReHo) achieved superior classification accuracy in distinguishing between PTSD and HCs, HCs and TECs, and PTSD and TECs [5]. The classification accuracy of this multimodal dataset was significantly higher than that of the GMV dataset (89.19% vs. 75.68%, 72% vs. 90%, and 59.46% vs. 67.57%). This highlights the limitations of sMRI and unimodal data in establishing precise biomarkers for diagnostic differentiation and treatment efficacy. The integration of multimodal data is essential to enhance the accuracy of differentiation and prediction beyond.

#### dMRI

Diffusion magnetic resonance imaging (dMRI) delineates axonal pathways in the human brain by assessing water molecule diffusion. Building upon diffusion-weighted imaging (DWI), diffusion tensor imaging (DTI) presents a more detailed examination of brain structures, facilitating a deeper understanding of changes in brain networks in PTSD patients. DTI has risen as the preferred approach for investigating the microstructural characteristics of neuronal tissues and reconstructing the structural connection pathways of the brain.

dMRI is increasingly utilized in PTSD research. A study by Dennis et al. [56] on a large sample revealed alterations in white matter tissue among PTSD patients, particularly affecting the hippocampal structure and interhemispheric connections. Mirjam et al. investigated white matter integrity in adolescents with PTSD, noting demyelination and myelination abnormalities in the corpus callosum [57]. They observed a correlation between anger scores and abnormalities in the left body of the corpus callosum, suggesting early-life trauma impacts corpus callosum integrity. Additionally, Meng et al. [58] studied white matter microstructure in Wenchuan earthquake survivors, finding that enhanced white matter integrity correlated with lower anxiety scores. This implies that improvements in white matter integrity could serve as crucial physiological markers for psychological resilience and stress recovery.

These studies underscore the importance of utilizing dMRI in clinical investigations of PTSD. dMRI is the only non-invasive imaging modality capable of providing individualized structural mapping and quantitative analysis of white matter tracts in the brain, elucidating the association between alterations in white matter integrity and PTSD symptomatology. There is a growing recognition of the utility of dynamic functional connectivity (dFC) in the brain as a biomarker for classifying and predicting psychiatric disorders [59]. Functional connectivity metrics are typically derived from both fMRI and dMRI data. Research indicates that employing multimodal approaches that integrate rs-fMRI and DTI may offer enhanced sensitivity to changes in brain connectivity, capture dFC patterns better, and emerge with superior predictive capabilities for PTSD recovery [60]. This work highlights the advantages of multimodal fusion techniques integrating dMRI with other imaging modalities over using dMRI alone. The integration of multimodal fusion technology in neuroimaging is poised to play a pivotal role in future studies investigating PTSD.

#### MR PWI

Perfusion-weighted imaging (PWI) using magnetic resonance (MR) is a valuable technique that assesses brain tissue function by detecting molecular movements indicative of microvascular perfusion and blood flow. Widely applied in scientific research and clinical practice, PWI is particularly useful in evaluating blood flow alterations in ischemic events such as acute stroke, vasospasm, and chronic steno-occlusive diseases. Continuous advancements have enhanced the accuracy of PWI, now comparable to nuclear medicine perfusion imaging. Its non-invasive nature makes it particularly promising for investigating subtle cerebral perfusion changes in psychiatric disorders. There are currently no monomodal PWI imaging studies on PTSD that have been retrieved.

### PET

Positron Emission Tomography (PET) is a laminar imaging technique that quantifies a broad spectrum of physiological, biochemical, and pharmacokinetic parameters by utilizing a radioactive tracer introduced into the body [61]. PET facilitates precise and non-destructive measurements of blood flow, glucose metabolism, neurotransmitter release, and drug delivery and uptake, making it instrumental in both neurobiological research and pharmaceutical exploration.

PET is a valuable tool for investigating PTSD by providing precise and sensitive neuromolecular information. Sophie et al. explored alterations in metabotropic glutamate receptor 5 (mGluR5) availability in subcortical regions (caudate nucleus, amygdala, hippocampus, and ventral striatum) of PTSD patients, suggesting a potential role of mGluR5 in PTSD pathogenesis [62]. Another study by Neumeister et al. [63] quantitatively assessed cannabinoid receptor 1 (CB1) availability in PTSD patients by PET, revealing abnormalities in the CB1 receptor-mediated cannabinoid signaling pathway linked to PTSD etiology. Additionally, Bhatt et al. [64] investigated brain immune regulation in PTSD patients, finding that peripheral immune activation correlated with inadequate activation of cerebral microglia. These PET studies elucidate mechanistic and functional neuronal changes in PTSD patients, enhancing our understanding of PTSD pathogenesis.

PET stands out as the most sensitive modality for functional imaging, capable of elucidating molecular and cellular mechanisms in patients with mental disorders during the early stages of molecular changes. Despite its advantages, PET has limitations, notably its invasive nature requiring the administration of radioactive substances and a low temporal resolution of 60–100 s, in contrast to fMRI which offers a resolution of approximately 2 s. Nonetheless, the integration of PET with MRI (PET+MRI) holds promise for advancing the development and assessment of novel therapeutics for PTSD. Recent studies propose the potential use of 3,4-methylenedioxymethamphetamine (MDMA) as an adjunct to psychotherapy for PTSD [65], with its mechanism of action involving alterations in neurotransmitters impacting affective memory circuits, subsequently influencing brain structure and function [66]. PET enables the evaluation of neuroplasticity changes at the molecular and cellular levels induced by drugs, while MRI can delineate functional consequences resulting from molecular and synaptic modifications. The synergy between PET and MRI technologies can provide a comprehensive understanding of drug mechanisms and therapeutic targets, facilitating the assessment of drug efficacy from micro to macro levels. Notably, this multimodal approach is also being leveraged to explore the therapeutic potential of hallucinogens in treating psychiatric disorders, including PTSD [67].

### SPECT

Single-Photon Emission Computed Tomography (SPECT) is a functional imaging technique that, like PET, involves administering a radiotracer to assess alterations in brain function by primarily measuring regional cerebral blood flow (rCBF).

SPECT imaging has been utilized to investigate the neural mechanisms associated with symptoms, treatment, and other facets of PTSD. Research using SPECT has revealed that, compared with healthy controls, individuals with PTSD exhibit reduced rCBF in the right thalamus, increased rCBF in the right parietal lobe [68], heightened rCBF in limbic brain regions [69, 70], and enhanced activation in the right insula and right superior/medial frontal gyrus [71], as well as dysfunction in the medial frontal gyrus compared with TEC. Regarding PTSD treatment, studies have demonstrated that, following cognitive restructuring therapy, individuals with PTSD show increased activation in the parietal, left hippocampal, thalamic, and left prefrontal cortex [71].

SPECT holds significant potential for diagnostic differentiation. In a study by Cyrus A. Raji et al. [72], SPECT was employed to distinguish between PTSD and individuals with mild traumatic brain injury (mTBI), achieving a differentiation accuracy of up to 94% at baseline. Being a widely utilized functional imaging modality, SPECT possesses distinc t advantages, including high usability and cost-effectiveness, enabling imaging of individuals with claustrophobia or metal implants. Nevertheless, SPECT is limited by lower image sensitivity, as well as reduced spatial and temporal resolution for deep brain measurements. Concerns also arise from the introduction of radioactive substances into the body. Notably, SPECT primarily offers insights into cerebral blood flow, providing only partial elucidation of the brain mechanisms underlying PTSD. To maximize the benefits of SPECT and address its limitations, integrating it into multimodal imaging studies is recommended to establish a comprehensive biomodel elucidating PTSD-related mechanisms in future research endeavors.

### EEG

EEG detects neural activity by capturing spontaneous rhythmic electrical signals on the scalp. Its precise temporal resolution and cost-effectiveness make it crucial for studying PTSD and its clinical implications.

A literature review examining the application of EEG in exploring the neural mechanisms of PTSD analyzed 34 studies. The review revealed a correlation between the severity of PTSD and P2 and P3 family event-related potentials, as well as alpha rhythm [73]. In a separate study, differences in EEG patterns were compared between PTSD patients and healthy individuals using dynamic Hurst analysis. The findings showed a significantly lower Hurst index in the F3 channel of PTSD patients compared to healthy controls, indicating that F3 could serve as a valuable neurophysiological marker for distinguishing between individuals with PTSD and those without [74].

The primary advantage of EEG technology lies in its high temporal accuracy, enabling precise identification of variations in micro-components of neural activity and a low cost. This feature has established EEG as a widely utilized objective measure for diagnosing PTSD. However, while EEG offers insights into brain activity, it remains an indirect measurement method lacking direct observation of neurons. Consequently, integrating EEG with other neuroimaging modalities is poised to emerge as a crucial approach in PTSD research. For instance, combining EEG with fMRI can yield brain imaging outcomes characterized by both high temporal precision and direct visualization of neural activity. This integrated approach holds promise for advancing understanding of the neural mechanisms underpinning PTSD, enhancing the accuracy of PTSD classification and diagnosis.

**Fig. 2:**
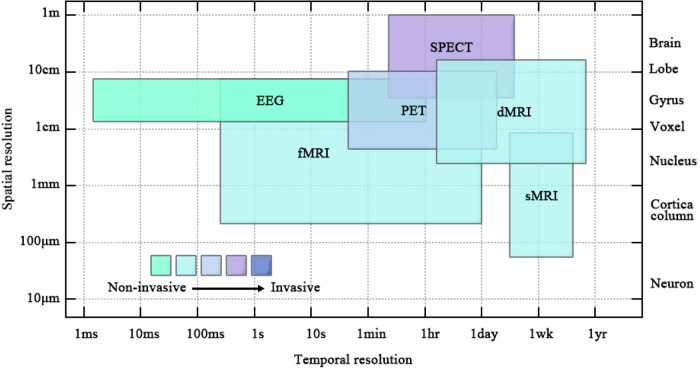
Spatiotemporal resolution of common unimodal brain images. This figure systematically compares various neuroimaging techniques in terms of their temporal-spatial resolution profiles and biological invasiveness characteristics, providing essential guidance for methodological selection in neuroscience research and clinical applications.

Overall, the spatiotemporal resolution and invasive of common brain imaging techniques are illustrated in Fig. 1 [51, 75] (Fig. 2). Functional imaging modalities include fMRI, PET, and SPECT. Among these, fMRI offers the finest spatial resolution, capable of sub-millimeter brain assessment, albeit with limited temporal resolution. PET and SPECT function by introducing radioactive substances to measure brain activity, with PET providing higher spatial resolution than SPECT and detecting biochemical brain changes. dMRI and sMRI are structural imaging methods, with dMRI focusing on white matter connectivity and sMRI on gray matter volume, which offering superior spatial resolution than dMRI. EEG boasts the highest temporal resolution but sacrifices spatial resolution due to its indirect measurement of brain activity through scalp electrodes. In conclusion, although each imaging modality presents distinct advantages and drawbacks, all of which are pivotal in ongoing research on the mechanisms, diagnosis, and treatment of PTSD.

## Multimodal brain imaging in PTSD diagnosis

Having examined the strengths and limitations of individual modalities, we now turn to discuss the clinical value of multimodal neuroimaging in PTSD diagnosis. The diagnosis of PTSD is currently facing several challenges. Firstly, it heavily relies on the subjective judgment of psychiatrists through structured or semi-structured interviews, leading to potential inefficiency and inconsistency in diagnosing PTSD. Distinguishing PTSD from other psychiatric disorders and TECS is a significant clinical issue. Additionally, PTSD frequently accompanies anxiety and depression, leading to inadequate treatment and affecting therapeutic results. As a result, addressing trauma-related cross-diagnosis poses a clinical difficulty. Identifying different subtypes of PTSD, such as the dissociative subtype and complex PTSD, poses another challenge in diagnosis. Moreover, trauma is a necessary but not sufficient condition for developing PTSD, as individual differences in personality traits, life experiences, and socio-cultural environments can influence psychological resilience. Therefore, predicting who will develop PTSD after trauma is difficult. It is crucial to identify biomarkers associated with susceptibility to PTSD for accurate diagnosis. Some scholars are using multimodal brain imaging to address these challenges and have made valuable progress.

Multimodal brain imaging is now essential for differentiating PTSD from other psychiatric disorders and TECs, greatly improving the precision of diagnosis. In a study, features extracted from sMRI and dMRI data effectively distinguished between trauma-exposed and non-trauma-exposed groups at different time points, with the amygdala emerging as a key marker [76]. A recent study combining sMRI, dMRI, and rs-fMRI revealed that modifications in the default mode network’s structure and function can effectively differentiate between PTSD and TEC. Additionally, the multimodal approach demonstrated a much higher discriminative power than individual imaging techniques [29]. Furthermore, a study utilizing sMRI and rs-fMRI techniques on PTSD patients, TECs, and HCs revealed that combining multimodal features yielded higher classification accuracies (67.57, 89.19, and 90%) when discriminating between PTSD and TECs, PTSD and HCs, or TECs and HCs, compared to using a single modality [5]. These studies collectively underscore the potential of integrating multimodal brain imaging data and machine learning in enhancing PTSD diagnosis. By leveraging different data modalities that capture distinct aspects, multimodal imaging can synergistically enhance classification performance, validate findings, and ultimately facilitate clinical diagnosis of PTSD.

Furthermore, multimodal neuroimaging has played a pivotal role in advancing transdiagnostic research in PTSD. Transdiagnosis involves pinpointing shared mechanisms among psychiatric disorders that may exist on a continuum. Utilizing multimodal brain imaging is crucial for validating transdiagnostic strategies for psychiatric disorders within the trauma spectrum. A recent study utilizing PET and multimodal MRI revealed that the prefrontal cortex neural circuits governing top-down cognition underlie trauma-related disorders across diagnostic categories [77]. These findings underscore the shared neural pathways between PTSD and other trauma-related emotional and cognitive disorders, providing a transdiagnostic view across the trauma spectrum.

Moreover, PTSD can be classified into distinct subtypes based on specific diagnostic criteria. For example, it can be categorized as the dissociative subtype of PTSD if dissociative symptoms are present. It can also be classified as complex PTSD based on the type of trauma experienced. However, there may be other subtypes of PTSD that have not been fully explored. Recently, Functional connectivity and anatomical connectivity features of the prefrontal cortex have been identified as biomarkers that can differentiate between the emotional numbing subtype and the hyperarousal subtype of [16]. Besides, Nicholson et al. [78] employed a multitask Gaussian process to analyze two data modalities. Their study successfully classified PTSD, the dissociative subtype of PTSD (PTSD + DS), and healthy individuals based on extracted features, demonstrating the potential of multimodal data in capturing distinctions between PTSD subtypes.

Multimodal brain imaging has also been utilized to identify genetic biomarkers for confirming PTSD diagnosis. A recent study integrated sMRI, MR PWI, and serum-extracted genetic data. The study revealed that variations in microRNA expression associated with the FK506-binding protein 5 gene were correlated with the severity of PTSD symptoms. These differences can explain changes in prefrontal brain structure and function as observed in brain imaging [79]. By combining genetic and multimodal brain imaging data, the study underscores the potential of these epigenetic biomarkers to complement PTSD diagnosis across molecular, brain, and behavioral domains. Furthermore, these findings suggest a foundation for developing mechanism-based preventive treatments for PTSD.

Multimodal neuroimaging can help identify biomarkers linked to vulnerability to PTSD. Post trauma anhedonia (PTA) is a symptom of PTSD that signifies dysfunction in post-traumatic reward circuits. In a study by Harnett et al. [80], the relationship between white matter microstructure, GMV, and PTA was examined. The findings suggest that the microstructure of the amygdala-prefrontal white matter junction may serve as a biomarker for susceptibility to PTA. Another study discovered that the frequency of microstates in the salience network correlates with PTA [81]. And a different multimodal neuroimaging study found a negative correlation between FA values in the bilateral prefrontal regions and voxel-mirrored homotopic connectivity (VMHC) in the parahippocampal gyrus and insula with CAPS scores. This suggests that these imaging findings could be markers for predisposition to PTSD [82]. These studies show that multimodal neuroimaging can forecast the development of PTSD by identifying biomarkers linked to susceptibility. This is crucial for clinical practice, as it allows for early detection and intervention in the early phases of the disorder, ultimately enhancing the outlook for individuals with PTSD.

**Fig. 3:**
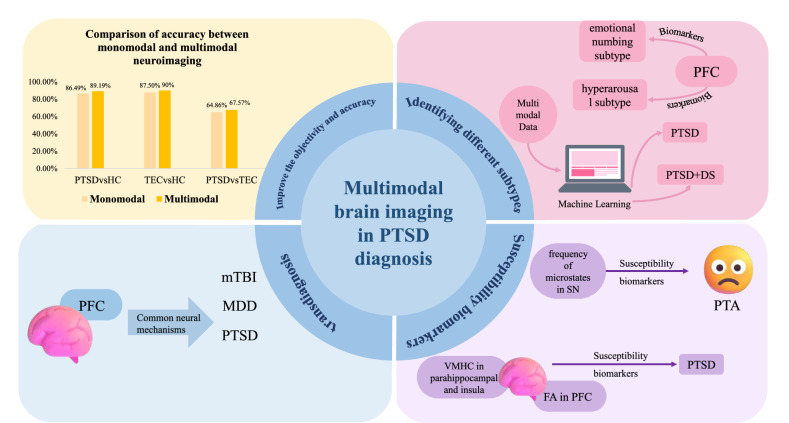
Multimodal brain imaging in PTSD diagnosis. Multimodal neuroimaging contributes to PTSD diagnosis through four primary aspects: enhancing diagnostic accuracy, investigating shared neurobiological mechanisms across diagnostic categories, identifying diagnostic-relevant neural targets, and differentiating PTSD subtypes. These contributions collectively advance our understanding of PTSD’s neural underpinnings and improve diagnostic precision in clinical practice.

In summary, multimodal neuroimaging has made significant advancements in improving the accuracy of differential diagnosis, aiding in the transdiagnostic approach to trauma-related psychiatric disorders, differentiating various subtypes of PTSD, and pinpointing biomarkers for PTSD diagnosis and susceptibility (Fig. 3). This type of imaging offers unique benefits for PTSD diagnosis by providing multi-dimensional data from different modalities that can enhance classification and prediction accuracy using machine learning techniques. Furthermore, multimodal neuroimaging can integrate neuro-molecular data from PET scans to identify biomarkers for susceptibility to PTSD, allowing clinicians to identify high-risk individuals early on and respond promptly, especially in the case of large-scale disasters. It is recommended that multimodal neuroimaging be more widely utilized in research to improve PTSD diagnostic capabilities.

## Multimodal brain imaging in PTSD treatment

Beyond diagnostic challenges, PTSD presents significant therapeutic complexities that warrant careful consideration. There are multiple international treatment guidelines for PTSD, with psychotherapy and pharmacotherapy being the main treatment approaches. Most guidelines recommend cognitive behavioral therapy (CBT) and selective serotonin reuptake inhibitors (SSRIs) as first-line treatments. Additionally, physical therapies like repetitive transcranial magnetic stimulation (rTMS) are becoming more common. However, PTSD treatment faces several challenges. Firstly, the mechanisms of action for many therapies are not fully understood, and only some patients respond to these treatments. It is unclear which patients are best suited for which therapies. Secondly, PTSD involves cognitive impairments and complex symptoms with unique triggering mechanisms, but the therapeutic targets for these symptoms are not fully explored. Lastly, PTSD is influenced by various complex factors and is a dynamically evolving condition. Current treatments are usually provided only during the chronic stage, neglecting other stages of the disorder. The treatment priorities for different developmental stages of PTSD are also unclear. There are other issues in PTSD treatment as well, but these challenges significantly impact treatment effectiveness and prognostic outcomes. Multimodal neuroimaging has provided insights into some of these issues.

Multimodal brain imaging is pivotal in elucidating the mechanisms underlying therapeutic interventions and assessing their effectiveness. In a study, Zhu et al. [7] employed multimodal MRI to forecast the therapeutic outcomes of equine-assisted therapy (EAT). Their results demonstrated that baseline connectivity levels of the caudate nucleus were predictive of the eventual efficacy of EAT. Moreover, EAT intervention ameliorated dysfunction in the reward system, encompassing cortico-striatal-thalamocortical circuits. Patients with PTSD often experience suicidal ideation due to the distress caused by their symptoms. Barredo et al. [8] investigated the efficacy and mechanism of transcranial magnetic stimulation (TMS) in reducing suicidal ideation through multimodal MRI analysis. The results suggest that TMS can reduce suicidal thoughts in patients by altering the functional connectivity between the dorsal striatum and frontal cortex. Additionally, the integrity of frontostriatal white matter may (WM) impact the functional connectivity response to TMS for suicide. These studies have shown how multimodal neuroimaging can help understand the mechanisms of PTSD treatment methods and provide a basis for interventions. However, they have not yet explained why different therapies have varying levels of effectiveness. Future research should also explore how multimodal neuroimaging can be used to investigate the variability in treatment outcomes for PTSD, in order to determine which patients would benefit most from which treatment plans and advance precision medicine.

In addition to understanding therapeutic mechanisms, the identification and validation of treatment targets represent a crucial aspect of PTSD intervention strategies. Identification of treatment targets for PTSD significantly influences therapeutic methods. Multimodal neuroimaging has provided insights into treatment targets for specific symptoms. Marin et al. [6] utilized fMRI and PET techniques to examine the link between trauma exposure, extinction learning, brain activity, and metabolism at rest. Their findings indicated a correlation between resting metabolism and functioning of fear circuits, PTSD symptoms, and ablative learning activation, notably in the amygdala. These results suggest that modulating brain metabolism in the resting state of PTSD patients could potentially improve their extinction learning deficits, offering avenues for therapeutic interventions such as targeted brain stimulation. One study found that the volume of the right superior parietal lobe and the density of WM integrity connected to this region are associated with avoidance symptoms in PTSD patients with adverse childhood experiences, suggesting the right superior parietal lobe as a valuable treatment target [83]. Another study indicated that PTSD patients exhibit impaired attentional control during emotional tasks, linked to deficits in the frontoparietal and limbic networks, as well as increased activation in the salience network. The anterior cingulate cortex may serve as a treatment target for attention deficits [37]. Insomnia and nightmares are hallmark symptoms of PTSD. Research found that sleep disturbances are associated with gray matter volume loss in anterior limbic/paralimbic and functional alterations in regions strongly linked to PTSD, such as the amygdala and hippocampus, suggesting potential targets for treating sleep disturbances in PTSD [84]. Confirmation of these targets can advance the development of PTSD treatments.

The progression of PTSD is a dynamic process, with brain regions associated with fear circuits exhibiting dynamic changes during the recovery phase [85]. Monitoring alterations in neural markers linked to symptom amelioration in PTSD recovery stages can elucidate the various phases of PTSD development and inform targeted interventions. A longitudinal study employing multimodal MRI scans revealed that the improvement of PTSD symptoms can be evaluated and predicted in the pre-recovery phase primarily based on the structural and connectivity features of the amygdala, and in the post-recovery phase based on changes in the structural and connectivity features of the OMPFC [76]. Simultaneously, amygdala features may serve as crucial neuromarkers for distinguishing between the pre-recovery and post-recovery phases of PTSD. These findings have implications for identifying the disease stage in clinical settings and tailoring treatments based on specific brain structural features at each stage.

**Fig. 4:**
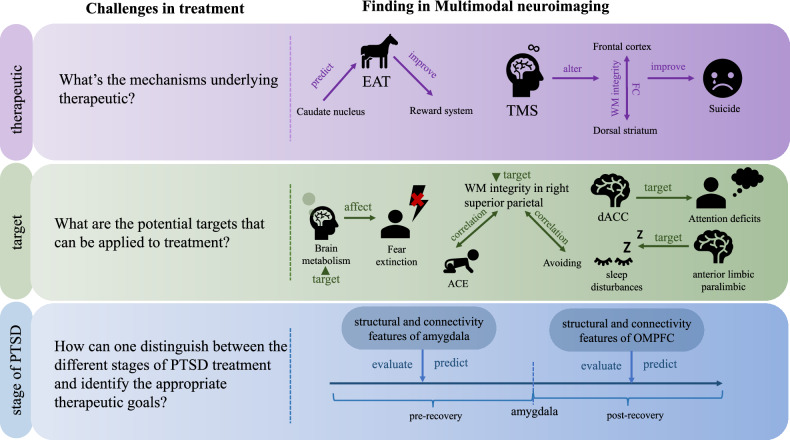
Multimodal brain imaging in PTSD treatment. Multimodal neuroimaging significantly contributes to PTSD treatment by elucidating therapeutic mechanisms, identifying potential treatment targets, and characterizing distinct developmental stages of PTSD. These applications facilitate the development of more targeted and effective therapeutic interventions for PTSD patients.

Overall, multimodal neuroimaging has made progress in exploring therapy mechanisms and identifying treatment targets in PTSD (Fig. 4). However, critical challenges remain unresolved, particularly regarding the mechanisms underlying interindividual variability in treatment response and the translation of identified targets into clinical applications. These limitations might impede the translation of multimodal neuroimaging research results into practical clinical applications, thus maintaining the current disconnect between scientific research and clinical practice. Future research could focus on these questions and leverage the potential of multimodal neuroimaging.

## Future of multimodal brain imaging

In summary, multimodal brain imaging serves similar purposes as single-modality imaging in the study and clinical application of PTSD. These include investigating PTSD-related brain mechanisms, identifying biomarkers for PTSD diagnosis and symptom amelioration, providing empirical evidence for clinical diagnosis and treatment of PTSD, and advancing the field of PTSD clinical diagnosis and treatment. However, multimodal brain imaging offers distinct advantages over single-modality approaches. Each single-modality imaging technique has limitations.For instance, MRI provides high spatial precision but low temporal resolution, while EEG offers high temporal resolution but low spatial accuracy. fMRI primarily maps brain function, sMRI focuses on brain structure, and dMRI examines white matter microstructure. As PTSD-related brain mechanisms are complex and involve dynamic interactions among neuroendocrine, structural, functional, and genetic factors in specific brain regions, these spatiotemporal changes collectively influence the onset and progression of PTSD. No single-modality brain image can fully capture the neural mechanisms underlying PTSD. Multimodal brain imaging involves integrating different modalities to combine the strengths and overcome the limitations of unimodal imaging. By leveraging complementary data from various modalities, a comprehensive understanding of PTSD can be constructed, enhancing researchers’ and clinicians’ insights into the mechanisms of disorders, refining diagnostic precision, and advancing personalized approaches to diagnosis and treatment.

The current application of multimodal brain imaging in PTSD research faces challenges. Most research on PTSD focuses on multimodal MRI, but there is limited utilization of cross-device data fusion. Most of PTSD multimodal brain imaging studies reviewed relied on multimodal MRI technology, with only a quarter explicitly mentioning cross-device synchronization for data acquisition. Challenges in cross-device synchronous data acquisition include potential interference between devices leading to artifacts. The integration of data from different devices acquired poses significant technical and analysis requirements. Existing techniques, such as EEG-MRI synchronized scanning and integrated TOF (time of flight)-PET/MR devices, provide a foundation for cross-device brain imaging data acquisition. Future studies are likely to develop cross-device synchronization techniques and advanced multimodal integrated devices. Exploring processing and data fusion methods tailored for cross-device data is also crucial to enhancing the application of multimodal brain imaging technology.

In addition to advancing cross-device integration technologies, the convergence of multimodal neuroimaging and machine learning holds significant promise for propelling the field forward. The integration of brain imaging with data-driven methodologies has been utilized to delineate brain-derived biotypes associated with PTSD. For instance, Ahrenholtz et al. [86] employed a data-driven approach to analyze fMRI data obtained from adult females with PTSD during fear conditioning and extinction tasks, leading to the identification of three distinct biotypes associated with the salience network, ventral visual network, and default mode network, respectively. These biotypes were found to correlate with different PTSD symptoms. Similarly, Stevens et al. [87] conducted a study involving clinical fMRI data from individuals exposed to traumatic events in the emergency department, utilizing hierarchical cluster analysis to reveal three brain functional activation patterns that could predict the severity of PTSD symptoms post-trauma. Nonetheless, the stability of biotypes identified in current brain imaging and data-driven investigations is questionable, as variations in identified biotypes across studies have been observed. This inconsistency may be attributed to the limited information provided by single-modal brain imaging data, hindering a comprehensive understanding of the trajectory of psychiatric illness development. The incorporation of multimodal imaging data into data-driven methodologies may offer a solution to identifying robust and reliable brain-derived biotypes, thereby enhancing efforts towards the prevention and personalized treatment of PTSD.

In fact, neuroimaging combined with machine learning techniques have been utilized in the categorization, diagnosis, and prognostication of PTSD. The research conducted by Zhang et al. showed that multimodal neuroimaging outperforms single-modal approaches in accurately distinguishing between PTSD, HC, and TEC [5]. This finding has also been supported by studies on various other mental disorders [88–90]. The richness and increased data volume of multimodal neuroimaging data confer a notable advantage in the application of machine learning for classification and prediction tasks. Multimodal neuroimaging extends its advantages beyond diagnostic efficiency, significantly propelling the advancement of personalized precision medicine. Although this technology has not yet addressed the critical challenge of determining optimal treatment strategies, and clinicians frequently encounter difficulties in selecting appropriate therapies based solely on initial clinical presentations - resulting in increased trial-and-error costs and suboptimal therapeutic outcomes - its comprehensive data acquisition capabilities offer promising solutions. By comparing pre-treatment characteristics between treatment responders and non-responders, researchers can identify predictive biomarkers and assess individualized therapeutic suitability. Furthermore, post-treatment comparative analyses can elucidate the mechanisms underlying treatment efficacy and uncover novel therapeutic targets, thereby facilitating the development of personalized treatment strategies.

## Conclusion

This review systematically examines the application of single-modality neuroimaging in both research and clinical settings for mental disorders, with a focus on PTSD. It highlights the significance of multimodality brain imaging in diagnosing and treating PTSD by elucidating brain mechanisms associated with the disorder, identifying biomarkers for diagnosis and symptom management, and providing empirical support for clinical interventions. The review underscores the advantages of multimodal brain imaging in offering a comprehensive understanding of PTSD mechanisms compared to single-modality approaches. Additionally, here we discuss future directions for research, suggesting the use of cross-device synchronous scanning technology, the development of integrated multimodal equipment, and optimization of data fusion techniques to enhance the application of multimodal brain imaging in PTSD. The potential role of integrating artificial intelligence with multimodal brain imaging is also proposed as a promising avenue for future investigation.
